# Falciparum Malaria in Febrile Patients at Sentinel Sites for Influenza Surveillance in the Central African Republic from 2015 to 2018

**DOI:** 10.1155/2020/3938541

**Published:** 2020-07-27

**Authors:** Romaric Nzoumbou-Boko, Brice Martial Yambiyo, Carine Ngoagouni, Ulrich Vickos, Alexandre Manirakiza, Emmanuel Nakouné

**Affiliations:** ^1^Laboratory of Parasitology, Institute Pasteur of Bangui, P.O. Box 923, Bangui, Central African Republic; ^2^Epidemiology Service, Institute Pasteur of Bangui, P.O. Box 923, Bangui, Central African Republic; ^3^Medical Entomology Service, Institute Pasteur of Bangui, P.O. Box 923, Bangui, Central African Republic; ^4^Virology Department, Institute Pasteur of Bangui, P.O. Box 923, Bangui, Central African Republic

## Abstract

Malaria is a major public health issue in the Central African Republic (CAR) despite massive scale-up of malaria interventions. However, no information is available on the incidence of malaria in febrile illness cases or on the distribution of malaria infection according to demographic characteristics, which are important indicators and valuable epidemiological surveillance tools. This study therefore aimed to characterize malaria in the network of sentinel sites set up for influenza surveillance. A retrospective analysis was conducted to explore the data from these sentinel sites from 2015 to 2018. The Paracheck-Pf® rapid diagnosis test kit was used to screen for malaria in febrile illness cases. A total of 3609 malaria cases were identified in 5397 febrile patients, giving an incidence rate of 66.8%. The age group of 1–4 years was the most affected by malaria (76.0%). Moreover, prevalence varied across different sentinel sites, with the Bossembele Health Center, located in a rural area, showing an incidence of 96%, the Saint Joseph Health Center in a semiurban area of Bangui showing an incidence of 75%, and the Bangui Pediatric Complex in an urban site with an incidence of only 44.6%. Malaria transmission was holoendemic over the four-year study period, and malaria incidence decreased from 2016 to 2018. The incidence of malaria coinfection with influenza was 6.8%. This study demonstrated clear microspatial heterogeneity of malaria. Malaria was consistently the most frequent cause of febrile illness. Including sites in different climate zones in the CAR will allow for a more representative study.

## 1. Introduction

In the Central African Republic (CAR), malaria is holoendemic and characterized by a high transmission rate, and the whole population (estimated at 4.7 million) is at risk for malaria infection [1]. Malaria is a major endemic illness and the leading cause of morbidity and mortality representing 50–60% of inpatient cases, with severe malaria estimated at 10% of all cases [1, 2]. In the CAR, children and pregnant women are the most vulnerable category of malaria patients, with malaria being the leading cause of death in children under 5 years [3]. *Plasmodium falciparum* is the prevailing species (99%) in Bangui, the capital city of the CAR [4]. A study carried out in Bangui identified *Anopheles gambiae* (63.2%) and *Anopheles funestus* (33%) as the primary malaria vectors [5].

Over the last 15 years, many initiatives have been carried out in the CAR, including the introduction of artemisinin-based combination therapies as the first-line treatment of unconfirmed malaria in 2005, the distribution of long-lasting insecticide-treated nets since 2010, and the introduction of a rapid diagnostic test (RDT) [6, 7]. However, despite efforts to control malaria through multiple schemes coordinated by the National Malaria Control Program (NMCP), malaria continues to be the major public health problem according to the scientific literature and the CAR country profile in the World Malaria Report. In 2010, two studies showed prevalence rates of 65.8% and 64.9%, respectively, in children at the Bangui Pediatric Complex (CPB) and in pregnant women in the Ouham-Pendé Prefecture [8, 9].

The 2019 World Malaria Report demonstrated that prevalence continued to increase in the CAR from 2015 to 2018. Prevalence rates were 68.9%, 71%, 73.3%, and 72.4% in 2015, 2016, 2017, and 2018, respectively, in the CAR, and in Africa, cases increased from 199 million to 213 million during the 2015–2018 period (an increase of 7%) [10]. Furthermore, these figures represent only the reported cases of malaria, which ignore the undetected and undiagnosed cases, especially in remote rural areas.

In the classification of malaria, the disease is called “simple” if the patient principally presents with fever, chills associated with headache, and muscle or joint pain, all of which are synonymous with an influenza-like illness [11, 12]. In other words, there are no “typical” malaria symptoms, and the clinical picture is therefore completely nonspecific, suggesting that a patient with a “flu” syndrome including at least one of the aforementioned symptoms may suffer from malaria unless proven otherwise [13]. In most cases in Africa, as in the CAR, fever is associated with malaria and vice versa: malaria is considered as the commonest cause of fever. RDTs have been developed for malaria to improve the management of fever especially among children [11, 14]. Importantly, to date, few data have been published on the differential diagnosis of flu syndromes and malaria [15, 16].

In the CAR, despite the NMCP strategic plan and outlook with goals to eliminate malaria by 2030, there are no sentinel sites for malaria surveillance activities, although disease surveillance is one of the elementary functions of public health systems [17]. Nevertheless, the Institut Pasteur in Bangui (IPB) and its Laboratory of Emerging Viruses and Zoonoses, the National Reference Center for influenza, set up a national influenza surveillance system in 2008 in collaboration with the Ministry of Health [18]. The surveillance system is based on the detection of influenza cases in a network of six sentinel sites. Here, we conducted an exploratory study to describe the cases of malaria from cases of influenza-like illnesses in these sentinel sites over the 2015–2018 period.

## 2. Methods

### 2.1. Type and Site of the Study

We conducted a retrospective study to analyze the malaria results in the sentinel site databases of the IPB influenza surveillance system from 2015 to 2018. The monitoring method used is described in [15]. The CAR, situated between 2°10′ and 11°N latitude, is an intertropical country with very diverse climates. Malaria transmission is holoendemic and occurs over the entire year, with peaks during the rainy season that occurs from April to November [19].

**Figure 1:**
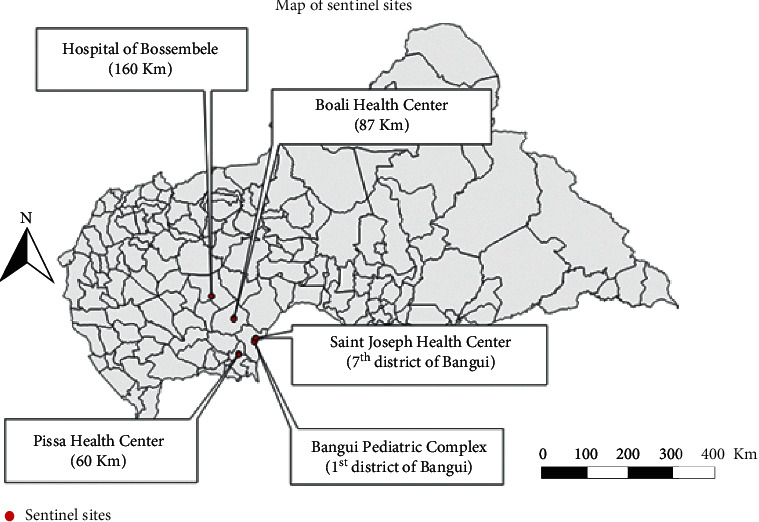
Map of sentinel sites for surveillance of influenza-like illness, Central African Republic, 2015–2018.

In 2008, two sentinel sites were opened in Bangui: at the CPB, located in the 1^st^ district (center of Bangui), and at the Saint Joseph Health Center in the 7^th^ district (southeast Bangui). In 2010, four other sites were opened within a maximum radius of 160 km around Bangui and located on the three national roads that serve the capital: the Pissa Health Center (west of Bangui), the Boali Health Center (north of Bangui), the Bossembele Hospital (northwest of Bangui), and Sibut Hospital (northeast of Bangui) (Figure 1). Due to the irregularity of the samples, the Sibut site was excluded from our study.

### 2.2. Study Population

The population consisted of febrile patients regardless of age or sex, visiting one of the sentinel sites for health care. The diagnosis of malaria has been systematically carried out as part of the surveillance of influenza since 2015 because malaria is the leading cause of fever in malarious areas [11, 20, 21]. We sought to compile information on patient residence (site), sex, and age.

### 2.3. Laboratory Screening for Malaria

The Paracheck-Pf® RDT (Orchid Biomedical Systems, India) was used. It is an immunochromatographic test based on the detection of *Plasmodium falciparum*-specific histidine-rich protein II (PfHRP-II) parasite antigens in lysed blood because this species is the dominant malaria parasite in the CAR.

### 2.4. Statistical Analyses of the Data

The data were recorded in an Access database (Microsoft Office 2016). Sentinel site maps were generated using the QGIS program (version 2.18.4), and the statistical analyses were carried out using STATA software (version 14; Stata Corp, College Station, Texas, USA).

Malaria-positive cases were estimated according to sex, age group (<1 year, 1–4 years, 5–14 years, 15–49 years, and ≥50 years), the sentinel site, influenza virus identification, and year. For each characteristic, the proportion of malaria cases was compared using the chi-squared test, and we determined the odds ratio (OR) to analyze the effect of each characteristic. *p* values <0.05 were considered to indicate statistical significance. We described trends in the proportion of malaria cases by month and by year.

## 3. Results

### 3.1. Characteristics of the Study Population

During the study period, 5397 febrile patients were enrolled, including 2570 males (47.62%) and 2822 females (52.38%), giving a sex ratio of 0.9 : 1. The average age was 11 years (range: 2 months to 78 years). The age group of 1–4 years was the most represented (36.4%) followed by 15–49-year-olds (23%). The Boali Health Center registered the most patients (31.0%) followed by the Saint Joseph Health Center in Bangui (18.8%) and Bossembele Hospital (18.8%). Years 2017 and 2018 showed the highest patient inclusion, with proportions of 29.0% and 32.0%, respectively.

### 3.2. Distribution of Malaria Infection by Demographic Characteristics

In total, among the 5397 febrile patients enrolled in the sentinel site network during the four-year study period, 3609 malaria cases were detected, corresponding to a prevalence of 66.8%. There were no differences according to sex (*p* value: 0.47). However, age groups showed significant differences: the age group of 1–4 years was the most affected by malaria (76.0%) followed by the age group of 5–14 years (73.0%). Under 1 year and over 50 years, prevalence was 60% and 67.0%, respectively. Finally, for the age group of 15–49 years, prevalence was 56%.

Prevalence also varied among sites. Bossembele Hospital showed a prevalence of 96% followed by the Saint Joseph Health Center with 75%. The Boali and Pissa Health Centers and CPB had prevalence rates of 62.0%, 52.0%, and 44.6%, respectively (Table 1).

The risk of malaria infection was highest for the age group of 1–4 years (OR = 2.2, 95% confidence interval (CI) [1.8, 2.6]; *p* value = 0.002) and for the age group of 5–15 years (OR = 1.7, 95% CI [1.34, 2.15]; *p* value = 0.001). For sentinel sites, the risk was highest at Bossembele Hospital (OR = 36.62, 95% CI [24.76, 53.26]; *p* value = 0.001) followed by the Saint Joseph Health Center (OR = 3.6, 95% CI [2.9, 5.52]; *p* value = 0.001).

**Figure 2:**
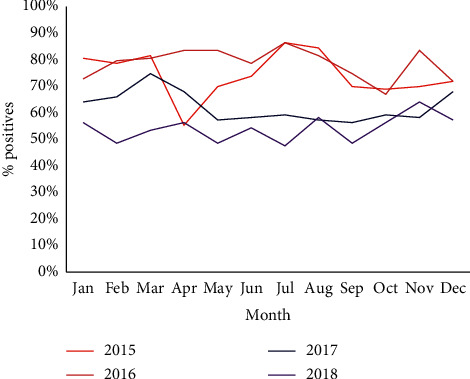
Monthly distribution of malaria incidence, Central African Republic, 2015–2018.

**Figure 3:**
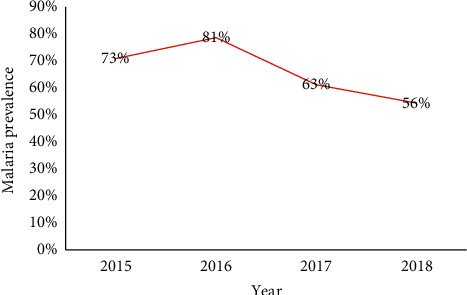
Yearly distribution of malaria, Central African Republic, 2015–2018.

The analysis of periodicity by month and year was almost homogeneous over the four-year study period (Figure 2). Furthermore, prevalence decreased over time from 2016 to 2018, dropping from 81% in 2016 to 63% in 2017 and finally to 56% in 2018 (Figure 3).

### 3.3. Comorbidity of Malaria and Influenza and Distribution of Influenza by Demographic Characteristics

The incidence of influenza over the four-year study period was estimated at 9.1% (491/5397 patients), including 367 (75%) cases of malaria-influenza coinfection. The incidence of malaria coinfection with influenza was 6.8%. The influenza strains associated with coinfection were A/H3N2 (43%), A/H1N1pdm09 (26%), and B (31%). We did not observe any significant differences in the distribution of the influenza strains according to the age group (*p* < 0.96), sex (*p* < 0.57), or sentinel sites (*p* < 0.1). However, analysis of the yearly trends showed significant differences (*p* < 0.001) with strain, with the A/H3N2 strain being predominant in 2016 (58.76%), the B strain predominating in 2017 (92.63%), and the A/H1N1Pdm09 strain predominating in 2018 (51.35%) (Table 2).

## 4. Discussion

Malaria prevalence according to demographic characteristics was assessed across a network of sentinel surveillance sites for influenza in the CAR between 2015 and 2018, a four-year follow-up period. Our results showed a downward trend for malaria incidence from 2016 to 2018 in the study sentinel sites. The 66.8% malaria incidence among the influenza-like cases showed that malaria is the most frequent cause of febrile illness in the CAR, unlike some African countries where malaria ranks second or third [11].

Our study confirms that children under 5 years of age are the most vulnerable group affected by malaria. Similar results have been obtained in Imo State, Nigeria [22]. Inversely, recent studies in western Kenya and in Franceville, Gabon, have reported a gradual shift in the at-risk-for-malaria age group to 7–9 and 5–14 years, respectively [21, 23, 24]. The risk of developing symptomatic malaria is effectively high in children under 5 years due to the immaturity of their immune systems [25, 26]. Adequate protective immunity (or partial immunity), antidisease immunity, and antiparasite immunity against symptomatic malaria and *P. falciparum* appear to be acquired more slowly and usually require repeated infections and intense exposure to the parasite [27, 28].

This shift in at-risk age groups indicates that the immuno-immaturity period has not changed and that children under five acquire malaria premunity earlier instead of gradually acquiring immunity. The control efforts for this age group should target decreased and interrupted exposure to reduce the number of malaria episodes, which cause the shift in the premunity period, and therefore the change in the at-risk group. However, our group of 15–49 years did not appear to be very at-risk, demonstrating acquired immunity in subjects inhabiting endemic areas after intense and uninterrupted exposure to malaria.

Statistical analyses revealed spatial variability in malaria prevalence. At the two sites located in the city of Bangui, the Saint Joseph Health Center (7^th^ district) showed a higher prevalence than the CPB (1^st^ district). Interestingly, the first entomological inventory of malaria vectors carried out in Bangui indicated an abundance of *A. gambiae sensu lato* and *A. funestus* in Ouango in the 7^th^ district [5]. The higher prevalence of these vectors in the 7^th^ district of Bangui can also be explained by the abundance of preferential breeding sites for mosquitos because in this district, various types of activities such as fish farming and brick making for the construction of houses, as well as the lack of plumbing, favor stagnant water. Furthermore, the 7^th^ district is considered a semiurban area, and similar results have been reported in Gabon, showing relatively high prevalence or transmission in semiurban areas compared with urban areas [29].

The Bossembele site, a rural area located at the edge of a savannah, showed high prevalence with 36 times of higher risk of transmission compared with the CPB. Again, this result echoes work in Gabon, showing a higher prevalence in rural areas than in semiurban and urban areas [29]. Bossembele is also situated at the intersection of national roads No. 1 and No. 3 that lead to Chad and Cameroon, suggesting that there is a relatively heavy population flow.

The difference in the frequency and regularity of malaria that is defined by the transmission rate at each site can also be due to epidemiological facies [30]. The network of sentinel sites included equatorial facies, tropical facies, urban facies, and the facies of local or transient events. However, in the absence of updated geographical data, particularly with regard to the climate and also entomological factors in this area, it is not possible to determine any relationship with malaria prevalence. Regarding Pissa, located in an area forming a part of the forest zone in the southern part of the CAR, our results indicate a relatively low risk of transmission.

Overall, our results highlight the microspatial heterogeneity of malaria in the CAR. A study conducted in Kenya showed that the spatial heterogeneity of malaria reduces the effectiveness of control strategies [31]. Therefore, it is also important to assess the specificity of each area in the context of the CAR to implement objective and effective interventions. Thus, sentinel sites should be set up in different climate zones of the CAR to assess spatial dynamics at the national level.

The monthly malaria prevalence was remarkably homogeneous, despite alternation between a rainy season (May–October) and a dry season (November–April). This lack of seasonal effect on transmission is surprising because the temperature and humidity conditions during the rainy season are favorable for the multiplication of immature malaria vectors (mosquito larvae) and therefore synonymous with high transmission. In contrast, studies in Burkina Faso, Ghana, and Kenya show seasonal transmission, varying with meteorological factors [32–34]. Nonetheless, the current climate change likely affects malaria dynamics, hence underlining the importance of carrying out small-scale studies throughout the country to assess the impact of microclimates.

Although the network of sentinel sites was set up for influenza, malaria is proving to be one of the major causes of febrile episodes at these sites. The prevalence of malaria coinfection with influenza, 6.8% (*n* = 5397) during the study period, is similar to a malaria-influenza coinfection study in Kenya, with a prevalence of 4–8% among children under 5 years of age [35]. Our data did not reveal any disparities in influenza strains according to the age group, sex, or sentinel site. In contrast, the prevalence of the A/H1N1Pdm09 strain was the highest in children under 5 years in the CAR network of sentinel sites during the 2010–2015 period [18]. The lack of literature on malaria-influenza coinfection does not allow us to assess the relevance of the malaria-influenza coinfection. Our study nonetheless illustrated that this network of sentinel sites can be used as syndromic epidemiological surveillance sites for several febrile diseases, such as arboviruses, in addition to malaria and influenza. Other infectious diseases such as salmonellosis and diarrheal diseases can also be monitored using the same operational resources.

In the case of malaria, the collection of dried blood spot samples at these sites will make it possible to not only determine the circulating *Plasmodium* species but also help characterize strains of *P. falciparum* and look for signatures of drug resistance included in the National Malaria Control Protocol.

## 5. Conclusion

Our analysis showed a relative decrease in malaria prevalence at selected influenza surveillance sites from 2015 to 2018. Malaria was consistently the most frequent cause of febrile illness. Transmission is still holoendemic. The age group of 1–4 years is at higher risk and shows high prevalence. Microspatial heterogeneity was observed in Bangui and in provincial cities. In addition, rural areas are more at risk and showed a higher prevalence than those in semiurban and urban areas. The study of malaria-influenza coinfection requires improved diagnosis of pathologies with febrile symptoms. The inclusion of sites in different climate zones in the CAR will allow for a more representative study.

## Figures and Tables

**Table 1:** Distribution of malaria infection according to demographic characteristics, Central African Republic, 2015–2018.

Characteristics	Malaria				
	Positive, *n* (%)	Negative, *n* (%)	OR	CI (95%)	*p* value
*Sex*					
M	1731 (67)	839 (33)			
F	1875 (66.44)	947 (33.56)			

*Age group*					
<1 year	750 (60)	504 (40)			
1–4 years	1497 (76)	465 (24)	2.2	[1.8, 2.6]	0.001^*∗∗∗*^
5–14 years	473 (73)	177 (27)	1.7	[1.34, 2.15]	0.001^*∗∗∗*^
15–49 years	697 (56)	548 (44)	0.75	[0.6, 0.9]	0.005^*∗∗∗*^
≥50 years	192 (67)	94 (33)	1.05	[0.7, 1.4]	0.73

*Location*					
Bangui Pediatric Complex	316 (44.57)	393 (55.43)			
Saint Joseph	758 (75)	258 (25)	3.62	[2.9, 5.52]	0.001^*∗∗∗*^
Boali	1042 (62)	632 (38)	2.33	[1.91, 2.84]	0.001^*∗∗∗*^
Bossembele	979 (96)	36 (4)	36.32	[24.76, 53.26]	0.001^*∗∗∗*^
Pissa	514 (52)	467 (48)	1.86	[1.49, 2.31]	0.001^*∗∗∗*^

*Influenza*					
Negative	3240 (69)	1473 (31)			
Positive	367 (75)	124 (25)			

*Year*					
2015	825 (76.53)	253 (23.47)			
2016	817 (81)	188 (18)			
2017	1004 (63)	580 (36)			
2018	963 (56)	767 (44)			

^*∗∗∗*^The significant difference according to age group and location

**Table 2:** Distribution of influenza virus infection according to demographic characteristics, Central African Republic, 2015–2018.

Characteristics	Influenza strain			
	H3N2, *n* (%)	H1N1Pdm09, *n* (%)	B, *n* (%)	*p* value
*Sex*				
M	102 (43.04)	64 (27)	67 (28.27)	0.57
F	107 (41.15)	64 (24.62)	87 (33.46)	

*Age group*				
<1 year	**34 (42.5)**	16 (20)	**30 (37.5)**	0.96
1–4 years	**75 (40.11)**	**50 (26.74)**	**58 (31.02)**	
5–14 years	**23 (34.85)**	**20 (30.3)**	**22 (33.33)**	
15–49 years	**58 (46.77)**	**32 (25.81)**	**33 (26.61)**	
≥ 50 years	19 (47.5)	10 (25)	11 (27.5)	

*Location*				
Bangui Pediatric Complex	12 (30.77)	8 (20.51)	19 (48.72)	0.1
Saint Joseph	**52 (40.94)**	**26 (20.47)**	**48 (37.8)**	
CS-Boali	**76 (42.46)**	**56 (31.28)**	**46 (25.7)**	
HBOS	**42 (46.67)**	**24 (26.67)**	**22 (24.44)**	
CS-Pissa	27 (43.55)	14 (22.58)	19 (30.65)	

*Year*				
2015	31 (41.56)	**31 (41.56)**	13 (16.88)	0.001^*∗∗∗*^
2016	**103 (58.76)**	20 (11.3)	**50 (28.81)**	
2017	7 (5)	0	**88 (95)**	
2018	**69 (45.62)**	**76 (51.35)**	12 (2.35)	

^*∗∗∗*^The significant difference according to year.

## Data Availability

The Microsoft Excel data used for this study are available from the corresponding author upon request.
